# Prediction of prognosis in elderly patients with chronic heart failure based on random survival forest

**DOI:** 10.3389/fcvm.2025.1613975

**Published:** 2025-09-05

**Authors:** Xuewu Song, Yuan Bian, Changyu Zhu, Ziyi Dong, Jinqi Li, Haiyan Gao

**Affiliations:** ^1^ Department of Pharmacy, Personalized Drug Research and Therapy Key Laboratory of Sichuan Province, Sichuan Provincial People’s Hospital, School of Medicine, University of Electronic Science and Technology of China, Chengdu, China; ^2^ Department of Nuclear Medicine, Sichuan Provincial People’s Hospital, School of Medicine, University of Electronic Science and Technology of China, Chengdu, China

**Keywords:** heart failure, elderly, prognosis, random survival forest, predict

## Abstract

**Background:**

There is a lack of tools to identify the risk of poor prognosis in elderly patients with chronic heart failure (CHF). This study aimed to develop a random survival forest (RSF) model to predict the prognosis of elderly CHF patients.

**Methods:**

The primary endpoint of this was all-cause mortality. The secondary endpoint was the combined outcome of unplanned readmissions and all-cause mortality. Patients were divided into a training set and a test set at a ratio of 7:3. We established and compared the performance of the RSF model with that of the New York Heart Association (NYHA) functional classes, left ventricular ejection fraction (LVEF) and B-type natriuretic peptide (BNP) level in evaluating the prognosis of elderly CHF patients. Harrell's C-index, decision curve analysis (DCA) and calibration curves were the main evaluation metrics for the model.

**Results:**

A total of 525 patients were enrolled. At a median follow-up of 60.1 (46.2, 63.5) months, 168 (32.0%) patients reached the primary endpoint and 219 (41.7%) patients reached the secondary endpoint. The C-index of the RSF model for predicting the primary endpoint was 0.747 in the training set and 0.714 in the test set. For the secondary endpoint, the C-index of the RSF model was 0.707 in the training set and 0.641 in the test set. DCA and calibration curves demonstrated that the RSF model showed good clinical usefulness and calibration.

**Conclusions:**

The RSF model showed good discrimination, clinical usefulness and calibration in predicting the prognosis of elderly CHF patients.

## 1 Introduction

Chronic heart failure (CHF) is a significant public health challenge, particularly among the elderly population (1). As the global demographic population continues to shift toward an aging society, the incidence and prevalence of CHF have increased alarmingly, leading to increased morbidity, healthcare costs, and mortality rates (2, 3). China has become one of the countries with the largest burden of heart failure worldwide. The post-discharge all-cause mortality of patients with heart failure was 13.7% at 1 year and 28.2% at 3 years (4). For patients aged 80 years and older, these rates are even higher, at 21.6% (1 year) and 43.2% (3 years), respectively (4).

Prognostic factors for elderly patients with heart failure encompass a range of clinical and demographic variables, including age, B-type natriuretic peptide (BNP) and N-terminal brain natriuretic peptide precursor (NT-proBNP) levels, New York Heart Association (NYHA) functional classes and left ventricular ejection fraction (LVEF) (5–9). Moreover, aging is often accompanied by a complex interplay of comorbidities and physiological changes, making the management and prognosis of heart failure in older adults particularly challenging (10). Given these complexities, effective predictive modeling is essential for guiding clinical decision-making and tailoring individualized treatment strategies.

Recent advancements in machine learning techniques have opened new avenues for developing predictive models that enhance our understanding of disease trajectories and outcomes in CHF patients (11). Among these techniques, the random survival forest (RSF) model has emerged as a powerful tool for analyzing time-to-event data in the presence of censoring (12). Unlike traditional survival analysis methods, RSF leverages the strengths of ensemble learning, allowing for the handling of high-dimensional data and nonlinear relationships without the need for parametric assumptions (13). RSF is currently the most widely used machine learning method for survival outcome prediction and has demonstrated strong performance in numerous clinical studies (14, 15). Its flexibility makes it particularly suitable for analyzing the complex clinical profiles of elderly CHF patients. However, to our knowledge, there have been no prognostic studies utilizing RSF in the context of elderly CHF patients.

Therefore, the purpose of this study was to establish an RSF model specifically designed to predict the prognosis of elderly CHF patients. Additionally, we compared the performance of the RSF model with that of traditional measures such as the NYHA class, LVEF and BNP levels in evaluating the prognosis of elderly patients with CHF.

## 2 Materials and methods

### 2.1 Study population

This retrospective study included elderly patients (aged ≥60 years) with CHF who visited Sichuan Provincial People's Hospital between January 2018 and January 2020. CHF was diagnosed according to the 2021 European Society of Cardiology (ESC) Guidelines for the Diagnosis and Treatment of Acute and Chronic Heart Failure, as well as the Chinese Guidelines for the Diagnosis and Treatment of Heart Failure (16, 17). Missing follow-up, in-hospital death, or serious data missing (defined as missing data ≥30%), and patients with tumors were excluded.

### 2.2 Follow-up and endpoints

Follow-up began on January 1, 2018, and the last follow-up was completed on June 1, 2024. The primary endpoint of the study was all-cause mortality. The secondary endpoint was defined as the combined outcome of unplanned readmissions and all-cause mortality during the entire follow-up period. Unplanned readmission was defined as any unscheduled hospitalization caused by heart failure exacerbation or related complications (e.g., arrhythmias, renal deterioration), as confirmed by physician assessment. This excluded scheduled elective surgeries, routine follow-ups or treatments (such as infusion of Levosimendan). And for patients with multiple readmissions, the first hospitalization record was analyzed.

### 2.3 Data collection and preprocessing

Patient data, including demographics, medical comorbidities, vital signs at discharge, laboratory tests, and discharge medications, were extracted from the hospital information system. Discharge medications were recorded as the number of medications (NOM) taken by the patient. All patients were classified according to NYHA classes I–IV. LVEF was measured via transthoracic echocardiography, cardiac magnetic resonance imaging, or computed tomography. Additionally, the left ventricular end-diastolic diameter (LV), left atrial diameter (LA), right ventricular end diastolic diameter (RV) and right atrial diameter (RA) were measured.

In this study, we excluded variables with missing values >30%, then used random forest to impute missing values for the remaining variables. This method allows retention of most clinically relevant variables while excluding those with excessive missing data that could distort results. Variable selection was performed via Boruta, an advanced method that can effectively identify and select the most relevant variables from large datasets, enhancing model performance and interpretability (18). One of the key features of Boruta is its ability to manage multicollinearity among the variables, which is crucial when dealing with real-world data where predictors may be correlated. In this study, the important variables selected by Boruta were subsequently used in RSF model development.

### 2.4 Development of prediction models

All patients were randomly divided into a training set and a test set at a 7:3 ratio. The training set was used to construct the RSF model, and the test set was used to evaluate the predictive performance. To compare the predictive performance of the RSF model with that of commonly used cardiac function indicators (NYHA class, LVEF and BNP level) in evaluating the prognosis of elderly CHF patients, we constructed 3 Cox proportional-hazards regression (Cox) models: Model 1 based on the NYHA class; Model 2 based on the NYHA class and LVEF; and Model 3 based on the NYHA class, LVEF and BNP level.

Model performance was evaluated in terms of discrimination, calibration, and clinical usefulness. Harrell's C-index was the primary evaluation metric. Decision curve analysis (DCA) was used to evaluate the clinical usefulness. Moreover, we analyzed the ability of different models to predict 3-year and 5-year outcomes in elderly patients with CHF. The time-dependent receiver operating characteristic (ROC) curve with the area under the curve (AUC) was the main evaluation metric. Calibration curves were used to evaluate the calibration performance of the RSF model. Patients were divided into low-risk and high-risk groups based on the median score of RSF predictions. Kaplan–Meier survival curves combined with log rank tests were used to illustrate the prognostic differences between these risk groups.

### 2.5 Statistical analysis

Normally distributed continuous variables were presented as the means and standard deviations (SD) and were compared using Student's *t*-test. Non-normally distributed continuous variables were presented as medians and interquartile ranges and were com-pared using Mann–Whitney *U*-test. Categorical variables were presented as counts and percentages. Statistical analysis was performed using the chi-square test or Fisher's exact test. A two-sided *P* value of <0.05 was considered statistically significant. All statistical analysis were performed using R version 4.4.0 (http://www.r-project.org/). The randomForestSRC package was used to construct the RSF model, while R packages including survival, survivalROC, timeROC, dcurves, rmda, and pec were employed for model performance evaluation.

## 3 Results

### 3.1 Baseline characteristics of patients

A total of 525 patients were enrolled in our study, comprising 232 (44.2%) males and 293 (55.8%) females. The median follow-up time was 60.1 (46.2, 63.5) months. During the follow-up period, 168 (32.0%) patients reached the primary endpoint and 219 (41.7%) patients reached the secondary endpoint. The median age of the participants was 76 (69–82) years, and the median length of stay was 8 (6–11) days. Patients with NYHA functional class III or IV symptoms at discharge accounted for 59.2%. The baseline characteristics of the patients are shown in Table 1.

**Table 1:** Baseline patient characteristics.

Characteristics	*N* = 525
Age, years	76 (69, 82)
Sex, *n* (%)	
Male	232 (44.2)
Female	293 (55.8)
Number of medications	7 (5, 9)
Follow-up time, months	60.1 (46.2, 63.5)
Length of stay, days	8 (6, 11)
Body mass index, kg/m^2^	23.8 (22.3, 25.2)
Heart rate, b.p.m	80 (70, 91)
Systolic blood pressure, mmHg	133 (118, 147)
Diastolic blood pressure, mmHg	74 (66, 85)
Left atrial diameter, mm	36 (34, 41)
Left ventricular end-diastolic diameter, mm	46 (42, 52)
Right atrium diameter, mm	45 (43, 48)
Right ventricular end diastolic diameter, mm	20 (19, 22)
LVEF, %	61 (49, 68)
Smoking status, *n* (%)	
No	330 (62.9)
Yes	195 (37.1)
Alcohol intake, *n* (%)	
No	391 (74.5)
Yes	134 (25.5)
NYHA Class, *n* (%)	
I or II	214 (40.8)
III or IV	311 (59.2)
COPD, *n* (%)	
No	455 (86.7)
Yes	70 (13.3)
Hypertension, *n* (%)	
No	192 (36.6)
Yes	333 (63.4)
Diabetes, *n* (%)	
No	366 (69.7)
Yes	159 (30.3)
Total triiodothyronine, nmol/L	1.20 (1.08, 1.35)
D-dimer, mg/L	0.61 (0.33, 1.19)
HDLC, mmol/L	1.20 (1.00, 1.41)
LDLC, mmol/L	2.16 (1.54, 2.82)
Albumin/globulin	1.3 (1.2, 1.6)
AST/ALT	1.3 (1.0, 1.9)
Alkaline phosphatase, U/L	81 (66, 95)
*γ*-glutamyl transpeptidase, U/L	34 (21, 63)
Cholinesterase, KU/L	6.6 (5.4, 7.9)
Total bilirubin, µmol/L	13.8 (9.7, 19.2)
eGFR, ml/min	75.5 (55.0, 89.3)
hsCRP, mg/L	3.3 (1.0, 11.9)
BNP, pg/ml	309.7 (112.2, 620.5)
White blood cell, 10^9^/L	6.73 (5.46, 8.58)
Basophil, 10^9^/L	0.03 (0.02, 0.04)
Red blood cell, 10^12^/L	4.19 (3.78, 4.63)
Hemoglobin, g/L	128 (114, 142)
Hematocrit	0.37 (0.22, 0.41)
RDW-SD, fL	45.1 (43.5, 47.3)
Primary endpoint, *n* (%)	
No	357 (68.0)
Yes	168 (32.0)
Secondary endpoint, *n* (%)	
No	306 (58.3)
Yes	219 (41.7)

LVEF, left ventricular ejection fraction; NYHA, New York Heart Association; COPD, chronic obstructive pulmonary disease; HDLC, high density lipoprotein cholesterol; LDLC, low density lipoprotein cholesterol; AST, aspartate aminotransferase; ALT, alanine aminotransferase; eGFR, estimated glomerular filtration rate; hsCRP, hypersensitive C-reactive protein; BNP, brain natriuretic pep-tide; RDW-SD, red blood cell distribution width-standard deviation.

### 3.2 Variable selection

**Figure 1:**
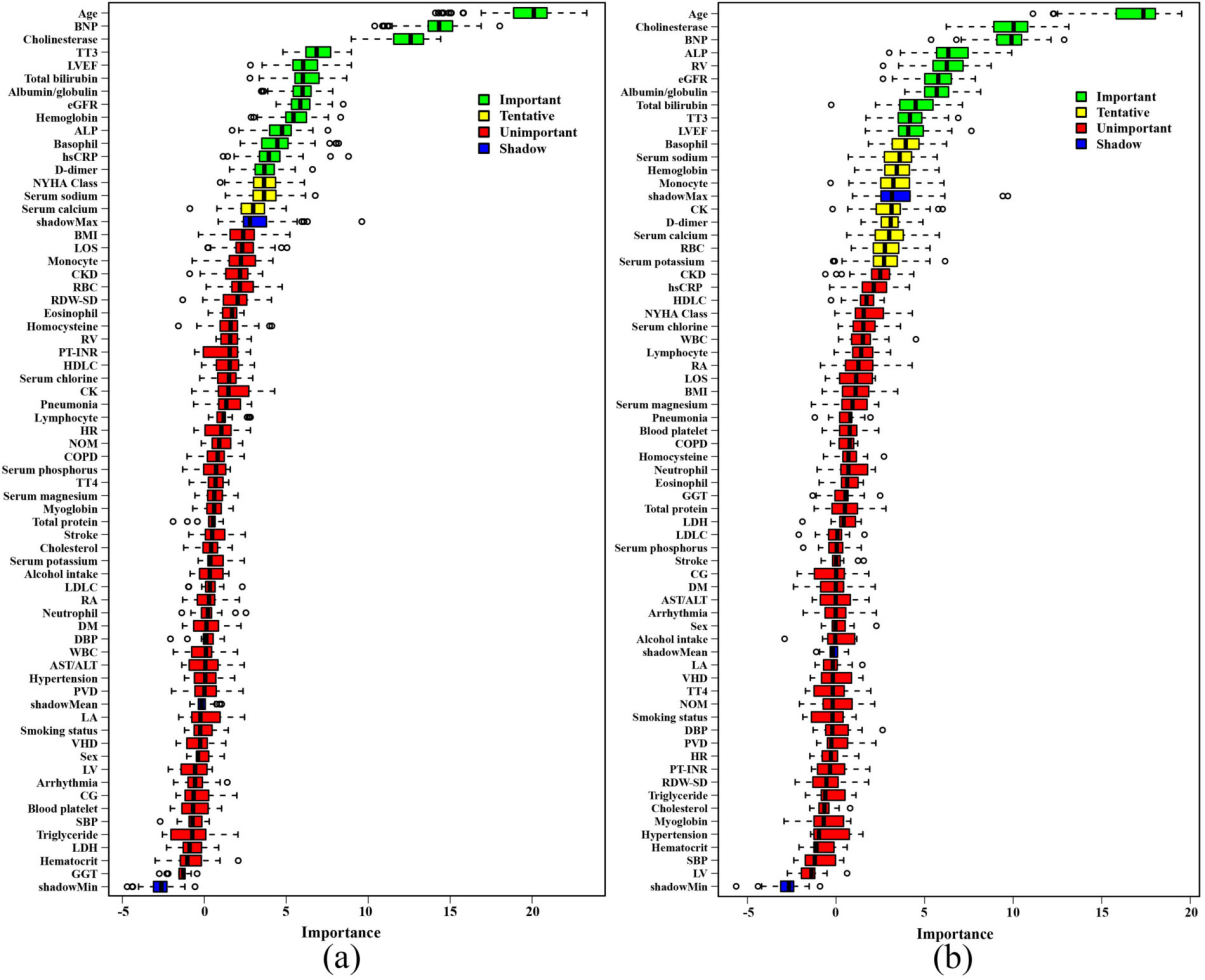
The results of boruta analysis. **(a)** Primary endpoint. **(b)** Secondary endpoint. BNP, brain natriuretic peptide; TT3, total triiodothyronine; LVEF, left ventricular ejection fraction; eGFR, estimated glomerular filtration rate; ALP, alkaline phosphatase; NYHA, New York Heart Association; BMI, body mass index; LOS, length of stay; CKD, chronic kidney disease; RBC, red blood cell; RDW-SD, red blood cell distribution width-standard deviation; RV, right ventricular end diastolic diameter; PT-INR, prothrombin time-international normalized ratio; HDLC, high density lipoprotein cholesterol; CK, creatine kinase; HR, heart rate; NOM, number of medications; COPD, chronic obstructive pulmonary disease; TT4, total thyroxine; LDLC, low density lipoprotein cholesterol; RA, right atrial diameter; DM, diabetes mellitus; DBP, diastolic blood pressure; WBC, white blood cell; AST, aspartate aminotransferase; ALT, alanine aminotransferase; PVD, peripheral vascular disease; LA, left atrial diameter; VHD, valvular heart disease; LV, left ventricular end-diastolic diameter; CG, chronic gastritis; SBP, systolic blood pressure; LDH, lactate dehydrogenase; GGT, *γ*-glutamyl transpeptidase.

A total of 65 variables were analyzed using the Boruta method. Thirteen variables were considered important in relation to all-cause mortality in elderly CHF patients, including age, BNP, cholinesterase, total triiodothyronine, LVEF, total bilirubin, albumin/globulin, estimated glomerular filtration rate (eGFR), hemoglobin, alkaline phosphatase, basophil, hypersensitive C-reactive protein (hsCRP) and D-dimer (Figure 1a). For the secondary endpoint, 10 important variables were age, cholinesterase, BNP, alkaline phosphatase, RV, eGFR, albumin/globulin, total bilirubin, total triiodothyronine and LVEF (Figure 1b). These variables were used to establish the RSF model.

### 3.3 Construction of the RSF model

**Figure 2:**
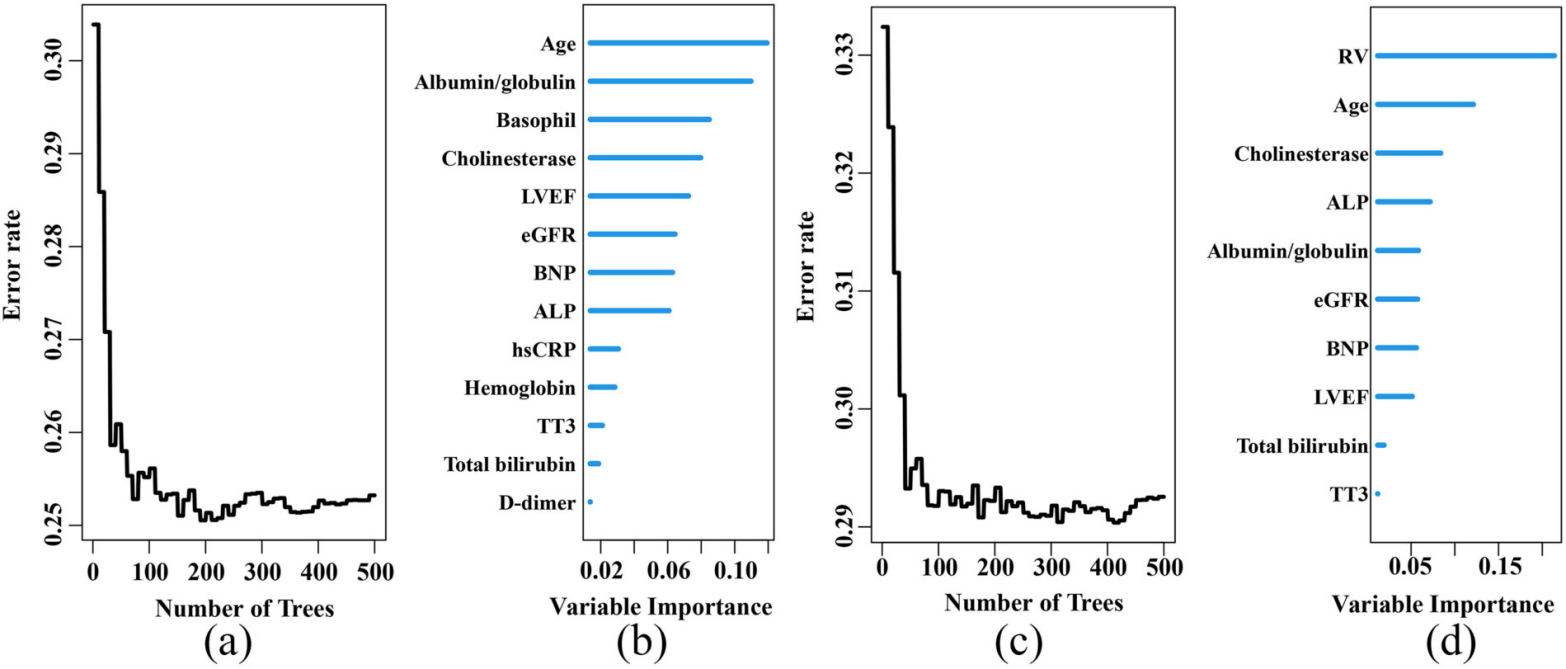
Construction of the RSF model to predict the prognosis of elderly patients with chronic heart failure in the training set. **(a)** Prediction error rates for the primary endpoint. **(b)** The variable importance plot for the primary endpoint. **(c)** Prediction error rates for the secondary endpoint. **(d)** The variable importance plot for the secondary endpoint. LVEF, left ventricular ejection fraction; eGFR, estimated glomerular filtration rate; BNP, brain natriuretic peptide; ALP, alkaline phosphatase; hsCRP, hypersensitive C-reactive protein; TT3, total triiodothyronine; RV, right ventricular end diastolic diameter.

An RSF model was constructed with 13 variables to predict all-cause mortality in elderly CHF patients. During the process of survival trees, the prediction error rate tended to be low and stable when the number of trees reached 200 (Figure 2a). When the number of survival trees reached 500, we analyzed the importance of each variable in predicting the outcome. As shown in Figure 2b, the 5 important variables for all-cause mortality in elderly CHF patients were age, albumin/globulin ratio, basophil count, cholinesterase level and LVEF.

Another RSF model was constructed using 10 variables to predict the combined out-come of unplanned readmissions and all-cause mortality in elderly CHF patients. When the number of survival trees reached 100, the prediction error rate tended to be low and stable (Figure 2c). When the number of survival trees reached 500, the 5 important variables were RV, age, cholinesterase level, alkaline phosphatase and albumin/globulin ratio (Figure 2d). The hyperparameter of the RSF model was shown in Supplementary Table S1.

### 3.4 The predictive performance of the models

Model discrimination was assessed using the Harrell's C-index. In the training set, the C-index values of the RSF model, Model 1, Model 2 and Model 3 in predicting the primary endpoint were 0.747, 0.630, 0.651 and 0.693, respectively. In the test set, the C-index values of the RSF model, Model 1, Model 2 and Model 3 were 0.714, 0.594, 0.623 and 0.658, respectively. For the secondary endpoint, the C-index values of the RSF model, Model 1, Model 2 and Model 3 in the training set were 0.707, 0.591, 0.595 and 0.645, respectively. In the test set, the C-index values of the RSF model, Model 1, Model 2 and Model 3 were 0.641, 0.552, 0.570 and 0.586, respectively.

**Figure 3:**
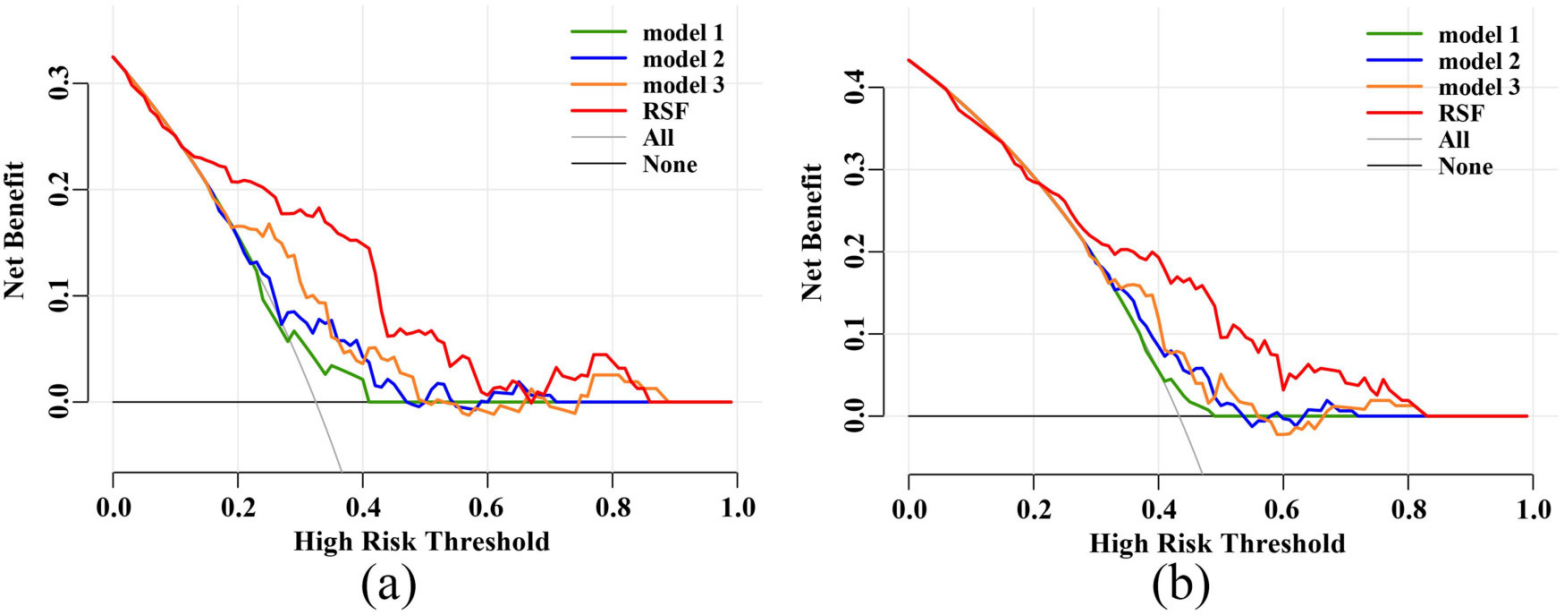
Decision curve analysis (DCA) in the test set. **(a)** DCA for the primary endpoint. **(b)** DCA for the secondary endpoint. RSF, random survival forest.

The DCA revealed that the RSF model provided a better net benefit than the other 3 models for predicting both primary and secondary endpoints in elderly CHF patients, in both the training and test sets (Supplementary Figure S1 and Figure 3).

**Figure 4:**
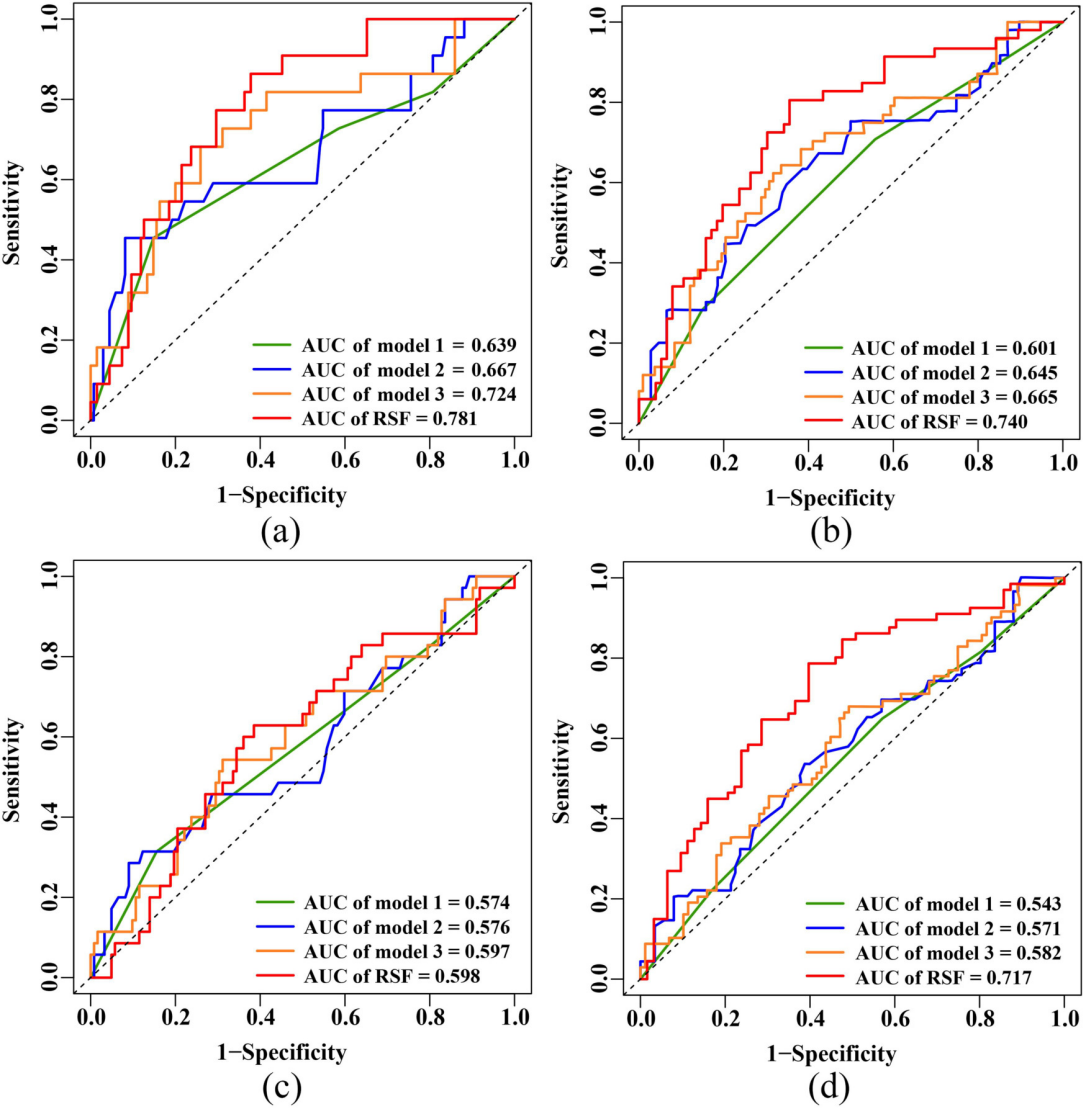
ROC curves in the test set. **(a)** 3-year primary endpoint. **(b)** 5-year primary endpoint. **(c)** 3-year secondary endpoint. **(d)** 5-year secondary endpoint. ROC, receiver operating characteristic; AUC, area under the curve; RSF, random survival forest.

For the 3-year primary endpoint, the AUCs of the RSF model, Model 1, Model 2 and Model 3 in the training set were 0.934, 0.632, 0.689 and 0.748, respectively (Supplementary Figure S2a). In the test set, the AUCs of the RSF model, Model 1, Model 2 and Model 3 were 0.781, 0.639, 0.667 and 0.724, respectively (Figure 4a). For the 5-year primary endpoint, the AUCs of the RSF model, Model 1, Model 2 and Model 3 in the training set were 0.912, 0.655, 0.671 and 0.715, respectively (Supplementary Figure S2b). In the test set, the AUCs of the RSF model, Model 1, Model 2 and Model 3 were 0.740, 0.601, 0.645 and 0.665, respectively (Figure 4b).

For the 3-year secondary endpoint, the AUCs of the RSF model, Model 1, Model 2 and Model 3 in the training set were 0.892, 0.585, 0.613 and 0.665, respectively (Supplementary Figure S2c). In the test set, the AUCs of the RSF model, Model 1, Model 2 and Model 3 were 0.598, 0.574, 0.576 and 0.597, respectively (Figure 4c). For the 5-year secondary endpoint, the AUCs of the RSF model, Model 1, Model 2 and Model 3 in the training set were 0.901, 0.611, 0.611 and 0.673, respectively (Supplementary Figure S2d). In the test set, the AUCs of the RSF model, Model 1, Model 2 and Model 3 were 0.717, 0.543, 0.571 and 0.582, respectively (Figure 4d).

**Figure 5:**
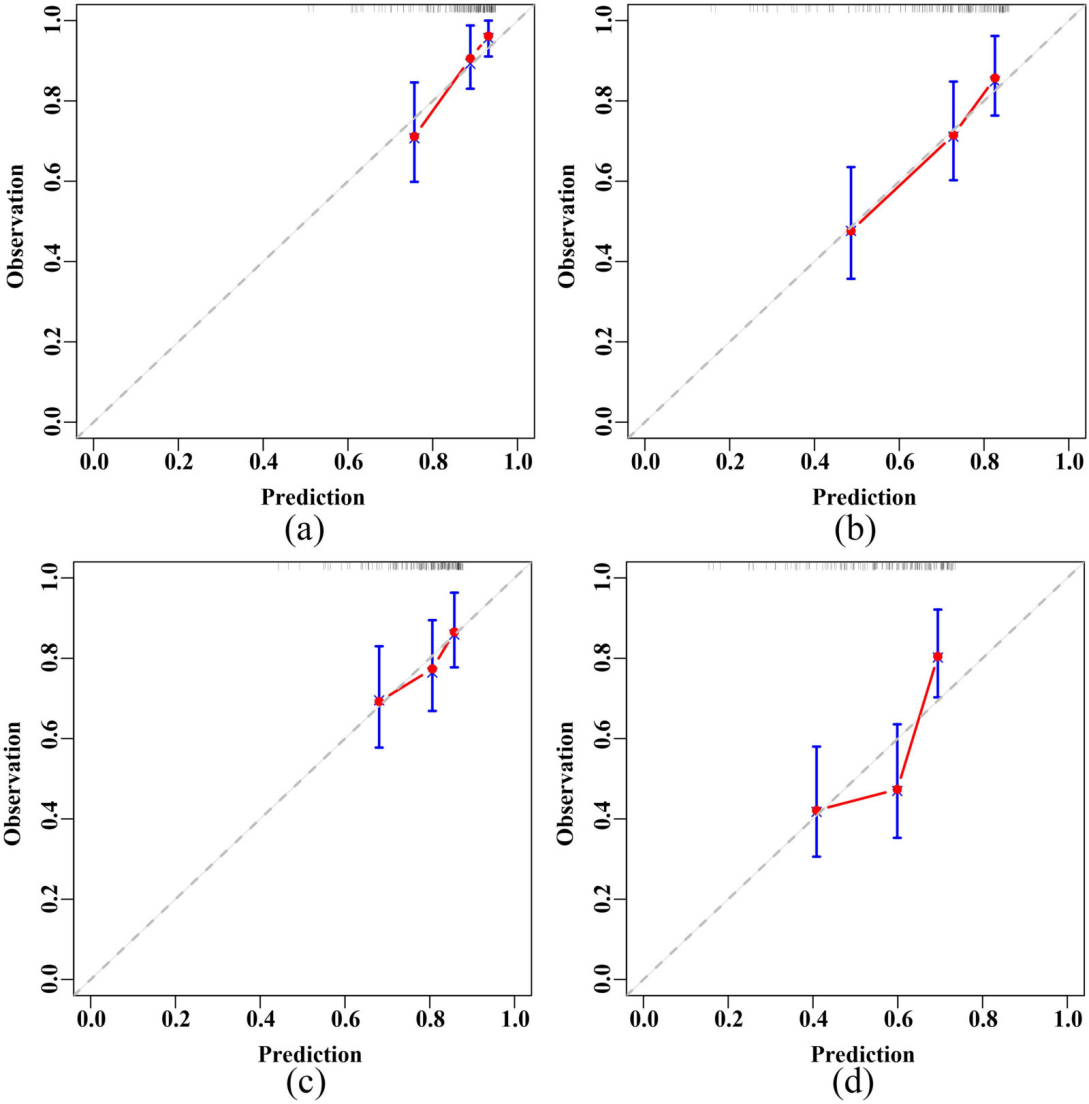
Calibration plots of the RSF model in the test set. **(a)** 3-year primary endpoint. **(b)** 5-year primary endpoint. **(c)** 3-year secondary endpoint. **(d)** 5-year secondary endpoint. RSF, random survival forest.

Calibration curves demonstrated the good calibration of RSF model in predicting both the 3-year and 5-year primary and secondary endpoints in elderly CHF patients, in both the training and test sets (Supplementary Figure S3 and Figure 5).

**Figure 6:**
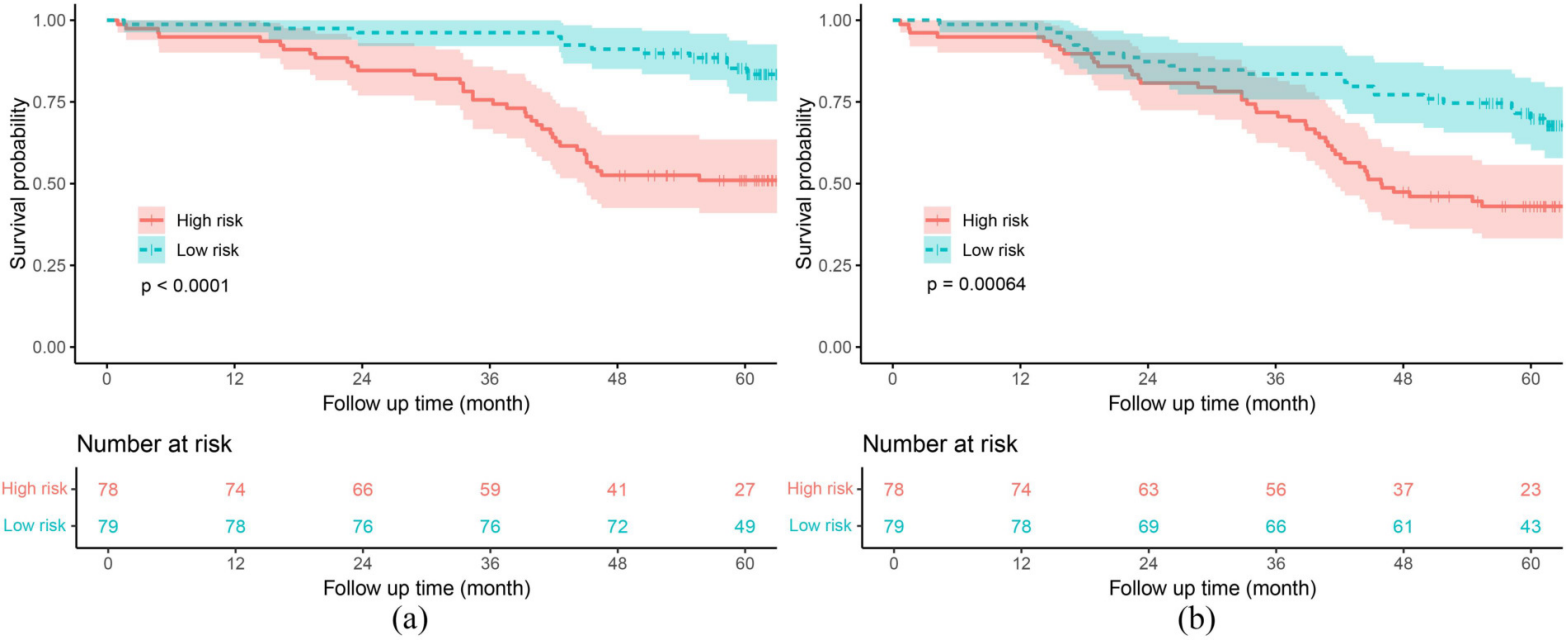
Kaplan–meier curves of the RSF model in the test set. **(a)** Primary endpoint. **(b)** Secondary endpoint. RSF, random survival forest.

Based on the median risk score predicted by the RSF model, patients were stratified into two risk groups (low-risk group vs. high-risk group). The Kaplan–Meier survival curves revealed that patients in the high-risk group had a worse prognosis for both the primary and secondary endpoints compared to those in the low-risk group, in both the training and test sets (both *P* < 0.001) (Supplementary Figure S4 and Figure 6).

## 4 Discussion

In this study, we constructed and compared the performance of the RSF model with 3 other basic models to predict the prognosis of elderly CHF patients. The results demonstrated that the RSF model exhibited good discrimination, calibration and clinical usefulness in predicting all-cause mortality, as well as consistent outcomes for unplanned readmissions and all-cause mortality among elderly CHF patients.

In recent years, many models have been developed to assess the in-hospital mortality rate of heart failure patients, neglecting the long-term survival outcomes, especially among the elderly (11, 19). The RSF model we established demonstrated good predictive performance for 3-year (AUC = 0.781) and 5-year (AUC = 0.740) mortality rates in elderly CHF patients. In China, the rate of unplanned readmissions is a critical indicator for evaluating hospital quality, and repeated readmissions further increase the medical bur-den on patients. Therefore, we aimed to establish a model to predict long-term unplanned readmissions and all-cause mortality in elderly heart failure patients. Unfortunately, while our RSF model could distinguish high-risk patients (Supplementary Figure S4b and Figure 6b), it had poor predictive performance for the secondary endpoint (C-index = 0.641). This may be attributed to the substantial variability among unplanned readmission patients, making it difficult for the model to accurately assess the long-term unplanned readmission situation in heart failure patients (20). Nonetheless, our findings are significant. The composite outcome of all-cause mortality and unplanned readmission holds significant clinical relevance as it comprehensively reflects both disease progression and acute deterioration, providing a more complete evaluation of patient outcomes than either measure alone. This combined endpoint better captures the substantial burden on patients, caregivers, and healthcare systems, particularly since unplanned readmission often precede mortality in this vulnerable population. Therefore, this patient-centered outcome serves as a robust indicator for evaluating management strategies, integrating both survival and healthcare utilization metrics. Moreover, to our knowledge, this is the first predictive model developed to assess long-term unplanned readmissions and all-cause mortality in elderly CHF patients. Furthermore, our results suggest that, compared with commonly used clinical heart function evaluation indicators (including the NYHA class, LVEF, and BNP level), the RSF model, which integrates multiple factors, has better predictive performance for long-term unplanned readmissions and all-cause mortality in elderly CHF patients.

Another key finding of our study is that age, the albumin/globulin ratio, cholinesterase, and LVEF are important predictive indicators of all-cause mortality or composite outcomes of unplanned readmissions and all-cause mortality in elderly CHF patients. In-creased age and decreased LVEF are associated with increased mortality in these populations. Serum ALB and GLB levels are part of routine biochemical tests, and the albumin to globulin ratio (AGR) serves as a biomarker of malnutrition and inflammation, reflecting the prognosis of heart failure patients (21, 22). Previous studies have demonstrated that the AGR is an independent predictor of mortality in patients with CHF (23), as well as an in-dependent predictor of cardiac events in women with heart failure with preserved ejection fraction (HFpEF) and heart failure-related rehospitalization (24). Our research revealed that including the albumin/globulin ratio among the risk factors for elderly CHF patients improved the prognostic value for clinical outcomes, including the composite result of unplanned readmissions and all-cause mortality.

Previous research has indicated that biomarkers such as AST, alanine aminotransferase, and alkaline phosphatase not only reflect liver cell damage but also serve as useful prognostic indicators for heart failure patients (25, 26). Sato Takamasa et al. (27) found that CHF patients with serum cholinesterase levels less than 240 U/L had significantly higher rates of cardiovascular death or readmission due to worsening heart failure. Similarly, Masayuki and his colleagues reported that the serum cholinesterase level outperformed other liver enzymes in the prediction of acute heart failure clinical outcomes (a composite of all-cause death and hospitalization for HF) (28). This finding is consistent with our findings. In elderly CHF patients, serum cholinesterase is an important predictive factor for both all-cause mortality and the composite outcomes of unplanned readmission and all-cause mortality. Interestingly, basophils are significant predictors of all-cause mortality in elderly heart failure patients, but are not significant for the composite outcome of un-planned readmissions and all-cause mortality. Patients experiencing acute heart failure or poor prognosis (1-year all-cause mortality) tend to have significantly lower eosinophil counts (29, 30). Our results suggest that eosinophils may also be potential biomarkers for all-cause mortality in elderly CHF patients. Additionally, in our study, the RV was found to be an important predictive factor for the composite outcome of unplanned readmissions and all-cause mortality in elderly CHF patients, but it was not an important predictor of all-cause mortality. The RV can predict the all-cause mortality rate in HFpEF patients (31), but research examining its prognostic significance in patients with reduced ejection fraction heart failure (HFrEF) is lacking.

Another strength of this study is that all predictive factors included in the RSF model are routine test indicators obtained during patients’ hospital admission, thereby not re-quiring additional burdens on the patients. Furthermore, in constructing the RSF model, we excluded variables that could not be determined by the Boruta algorithm (indicated as yellow variables in Figure 1). Although including these variables may improve the model's predictive performance, clinicians believe that a simpler model with fewer variables can enhance the model's clinical practicality and convenience.

However, our study has several limitations. First, it was conducted at a single center using a retrospective design. The sample size was relatively small. These factors may limit the generalizability of our findings. The small sample size also prevents us from performing detailed subgroup analyses. Second, our medication data were incomplete as we lacked information on dosage and patient adherence. This simplification may have reduced the model's predictive accuracy. Third, external validation is necessary. Future studies should involve multiple centers and include larger patient cohorts. Such studies would help confirm our results and improve their clinical applicability. Fourth, although we assessed all-cause mortality in elderly CHF patients, we were unable to evaluate mortality specifically due to cardiovascular diseases or heart failure. Finally, the lack of external validation may limit the generalization of our findings. Before clinical application, a multi-center prospective assessment and validation of the model are needed.

## 5 Conclusions

In this study, we established and compared the performance of the RSF model with that of the NYHA class, LVEF and BNP level in evaluating the prognosis of elderly CHF patients. The results demonstrated that the RSF model can effectively identify the elderly CHF patients at a high risk for poor prognosis.

## Data Availability

The raw data supporting the conclusions of this article will be made available by the authors, without undue reservation.

## References

[1] Khan MS Shahid I Bennis A Rakisheva A Metra M Butler J. Global epidemiology of heart failure. Nat Rev Cardiol. (2024) 21:717–34. 10.1038/s41569-024-01046-6 38926611

[2] Wang SY Valero-Elizondo J Ali HJ Pandey A Cainzos-Achirica M Krumholz HM Out-of-Pocket annual health expenditures and financial toxicity from healthcare costs in patients with heart failure in the United States. J Am Heart Assoc. (2021) 10:e022164. 10.1161/jaha.121.022164 33998273 PMC8483501

[3] Lan T Liao YH Zhang J Yang ZP Xu GS Zhu L Mortality and readmission rates after heart failure: a systematic review and meta-analysis. Ther Clin Risk Manag. (2021) 17:1307–20. 10.2147/tcrm.S340587 34908840 PMC8665875

[4] Wang H Li Y Chai K Long Z Yang Z Du M Mortality in patients admitted to hospital with heart failure in China: a nationwide cardiovascular association database-heart failure centre registry cohort study. The Lancet. Global Health. (2024) 12:e611–e22. 10.1016/s2214-109x(23)00605-8 38485428

[5] Sciomer S Moscucci F Salvioni E Marchese G Bussotti M Corrà U Role of gender, age and BMI in prognosis of heart failure. Eur J Prev Cardiol. (2020) 27:46–51. 10.1177/2047487320961980 33238736 PMC7691623

[6] Jia X Al Rifai M Hoogeveen R Echouffo-Tcheugui JB Shah AM Ndumele CE Association of long-term change in N-terminal pro-B-type natriuretic peptide with incident heart failure and death. JAMA cardiology. (2023) 8:222–30. 10.1001/jamacardio.2022.5309 36753229 PMC9909572

[7] Verma D Nath RK Pandit N Rahatekar P Vatsa D Bhutani M. Prognostic utility of B-type natriuretic peptide and 6-min walk test in patients with acute decompensated heart failure. Indian Heart J. (2024) 76:291–96. 10.1016/j.ihj.2024.07.011 39069072 PMC11451414

[8] Briongos-Figuero S Estévez A Pérez ML Martínez-Ferrer JB García E Viñolas X Prognostic role of NYHA class in heart failure patients undergoing primary prevention ICD therapy. ESC heart Failure. (2020) 7:279–83. 10.1002/ehf2.12548 31823514 PMC7083467

[9] Lam CSP Gamble GD Ling LH Sim D Leong KTG Yeo PSD Mortality associated with heart failure with preserved vs. Reduced ejection fraction in a prospective international multi-ethnic cohort study. Eur Heart J. (2018) 39:1770–80. 10.1093/eurheartj/ehy005 29390051

[10] Triposkiadis F Xanthopoulos A Parissis J Butler J Farmakis D. Pathogenesis of chronic heart failure: cardiovascular aging, risk factors, comorbidities, and disease modifiers. Heart Fail Rev. (2022) 27:337–44. 10.1007/s10741-020-09987-z 32524327

[11] Segar MW Hall JL Jhund PS Powell-Wiley TM Morris AA Kao D Machine learning-based models incorporating social determinants of health vs traditional models for predicting in-hospital mortality in patients with heart failure. JAMA cardiology. (2022) 7:844–54. 10.1001/jamacardio.2022.1900 35793094 PMC9260645

[12] Dietrich S Floegel A Troll M Kühn T Rathmann W Peters A Random survival forest in practice: a method for modelling complex metabolomics data in time to event analysis. Int J Epidemiol. (2016) 45:1406–20. 10.1093/ije/dyw145 27591264

[13] Wang H Zhou L. Random survival forest with space extensions for censored data. Artif Intell Med. (2017) 79:52–61. 10.1016/j.artmed.2017.06.005 28641924

[14] Zhang L Huang T Xu F Li S Zheng S Lyu J Prediction of prognosis in elderly patients with sepsis based on machine learning (random survival forest). BMC Emerg Med. (2022) 22:26. 10.1186/s12873-022-00582-z 35148680 PMC8832779

[15] Bohannan ZS Coffman F Mitrofanova A. Random survival forest model identifies novel biomarkers of event-free survival in high-risk pediatric acute lymphoblastic leukemia. Comput Struct Biotechnol J. (2022) 20:583–97. 10.1016/j.csbj.2022.01.003 35116134 PMC8777142

[16] McDonagh TA Metra M Adamo M Gardner RS Baumbach A Böhm M 2021 ESC guidelines for the diagnosis and treatment of acute and chronic heart failure: developed by the task force for the diagnosis and treatment of acute and chronic heart failure of the European Society of Cardiology (ESC). with the special contribution of the heart failure association (HFA) of the ESC. Eur J Heart Fail. (2022) 24:4–131. 10.1002/ejhf.2333 35083827

[17] Zhang SY. Chinese guidelines for the diagnosis and treatment of heart failure 2024. J Geriatr Cardiol. (2025) 22:277–331. 10.26599/1671-5411.2025.03.002 40351394 PMC12059564

[18] Zhou H Xin Y Li S. A diabetes prediction model based on boruta feature selection and ensemble learning. BMC Bioinformatics. (2023) 24:224. 10.1186/s12859-023-05300-5 37264332 PMC10236811

[19] Ma M Hao X Zhao J Luo S Liu Y Li D. Predicting heart failure in-hospital mortality by integrating longitudinal and category data in electronic health records. Med Biol Eng Comput. (2023) 61:1857–73. 10.1007/s11517-023-02816-z 36959414

[20] Au AG McAlister FA Bakal JA Ezekowitz J Kaul P van Walraven C. Predicting the risk of unplanned readmission or death within 30 days of discharge after a heart failure hospitalization. Am Heart J. (2012) 164:365–72. 10.1016/j.ahj.2012.06.010 22980303

[21] Armentaro G Condoleo V Pastura CA Grasso M Frasca A Martire D Prognostic role of serum albumin levels in patients with chronic heart failure. Intern Emerg Med. (2024) 19:1323–33. 10.1007/s11739-024-03612-9 38776047 PMC11364577

[22] Li K Fu W Bo Y Zhu Y. Effect of albumin-globulin score and albumin to globulin ratio on survival in patients with heart failure: a retrospective cohort study in China. BMJ open. (2018) 8:e022960. 10.1136/bmjopen-2018-022960 29982222 PMC6042582

[23] Niedziela JT Hudzik B Szygula-Jurkiewicz B Nowak JU Polonski L Gasior M Albumin-to-globulin ratio as an independent predictor of mortality in chronic heart failure. Biomark Med. (2018) 12:749–57. 10.2217/bmm-2017-0378 29865856

[24] Otaki Y Shimizu M Watanabe T Tachibana S Sato J Kobayashi Y Albumin-to-globulin ratio predicts clinical outcomes of heart failure with preserved ejection fraction in women. Heart Vessels. (2022) 37:1829–40. 10.1007/s00380-022-02087-y 35596031

[25] Nikolaou M Parissis J Yilmaz MB Seronde MF Kivikko M Laribi S Liver function abnormalities, clinical profile, and outcome in acute decompensated heart failure. Eur Heart J. (2013) 34:742–9. 10.1093/eurheartj/ehs332 23091203

[26] Yamashita M Kamiya K Hamazaki N Nozaki K Uchida S Maekawa E Predictive value of cholinesterase in patients with heart failure: a new blood biochemical marker of undernutrition. Nutr Metab Cardiovasc Dis. (2023) 33:1914–22. 10.1016/j.numecd.2023.06.005 37500349

[27] Sato T Yamauchi H Suzuki S Yoshihisa A Yamaki T Sugimoto K Serum cholinesterase is an important prognostic factor in chronic heart failure. Heart Vessels. (2015) 30:204–10. 10.1007/s00380-014-0469-8 24463844

[28] Shiba M Kato T Morimoto T Yaku H Inuzuka Y Tamaki Y Serum cholinesterase as a prognostic biomarker for acute heart failure. Eur Heart J Acute Cardiovasc Care. (2021) 10:335–42. 10.1093/ehjacc/zuaa043 33580775

[29] Yilmaz R Yagin FH Colak C Toprak K Abdel Samee N Mahmoud NF Analysis of hematological indicators via explainable artificial intelligence in the diagnosis of acute heart failure: a retrospective study. Front Med (Lausanne). (2024) 11:1285067. 10.3389/fmed.2024.1285067 38633310 PMC11023638

[30] Polat N Yıldız A Bilik MZ Aydın M Acet H Kaya H The importance of hematologic indices in the risk stratification of patients with acute decompensated systolic heart failure. Turk Kardiyol Dern Ars. (2015) 43:157–65. 10.5543/tkda.2015.76281 25782120

[31] Parrinello G Torres D Buscemi S Di Chiara T Cuttitta F Cardillo M Right ventricular diameter predicts all-cause mortality in heart failure with preserved ejection fraction. Intern Emerg Med. (2019) 14:1091–100. 10.1007/s11739-019-02071-x 30895427

