# Supplementary material for: Prediction of prognosis in elderly patients with chronic heart failure based on random survival forest
Source: Front Cardiovasc Med. 2025 Sep 5;12:1613975. doi: 10.3389/fcvm.2025.1613975 (PMC12446311; doi:10.3389/fcvm.2025.1613975)

Supplementary Material

# Supplementary Tables

**Table S1.** Hyperparameter of the RSF model

| Hyperparameter | Primary endpoint | Secondary endpoint |
| --- | --- | --- |
| Number of trees | 500 | 500 |
| Forest terminal node size | 15 | 15 |
| Average no. of terminal nodes | 18.32 | 20.088 |
| No. of variables tried at each split | 4 | 4 |
| Total no. of variables | 13 | 10 |
| Resampling used to grow trees | swor | swor |
| Resample size used to grow trees | 233 | 233 |
| Splitting rule | Logrank random | Logrank random |
| Number of random split points | 10 | 10 |

# Supplementary Figures

**Figure S1.** Decision curve analysis (DCA) in the training set. (a) DCA for primary endpoint. (b) DCA for secondary endpoint. RSF, random survival forest.


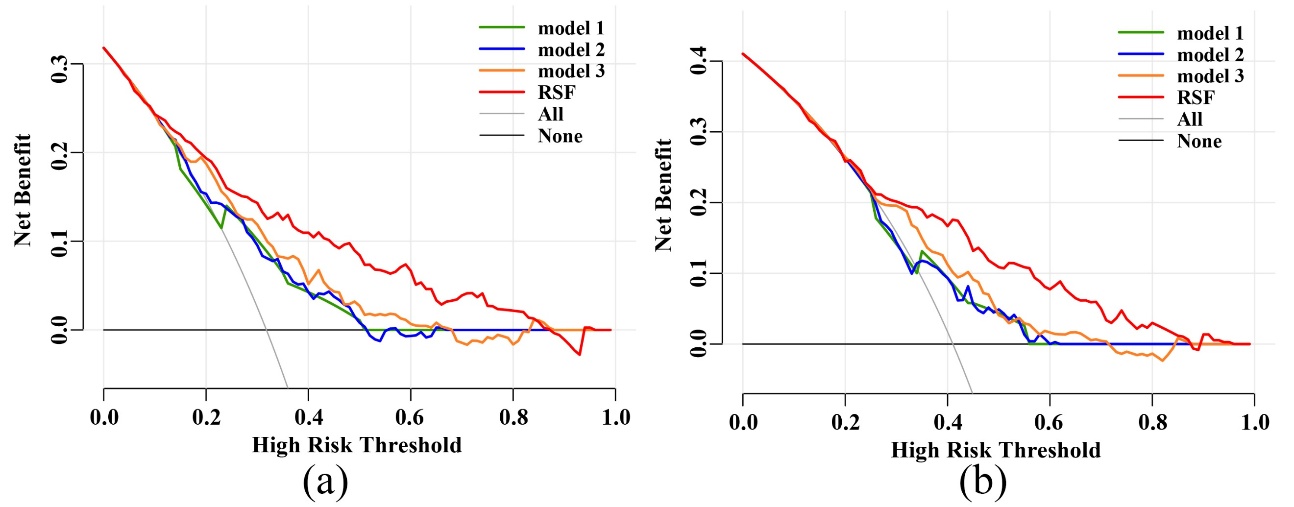


**Figure S2.** ROC curves in the training set. (a) 3-year primary endpoint. (b) 5-year primary endpoint. (c) 3-year secondary endpoint. (d) 5-year secondary endpoint. ROC, receiver operating characteristic; AUC, area under the curve; RSF, random survival forest.


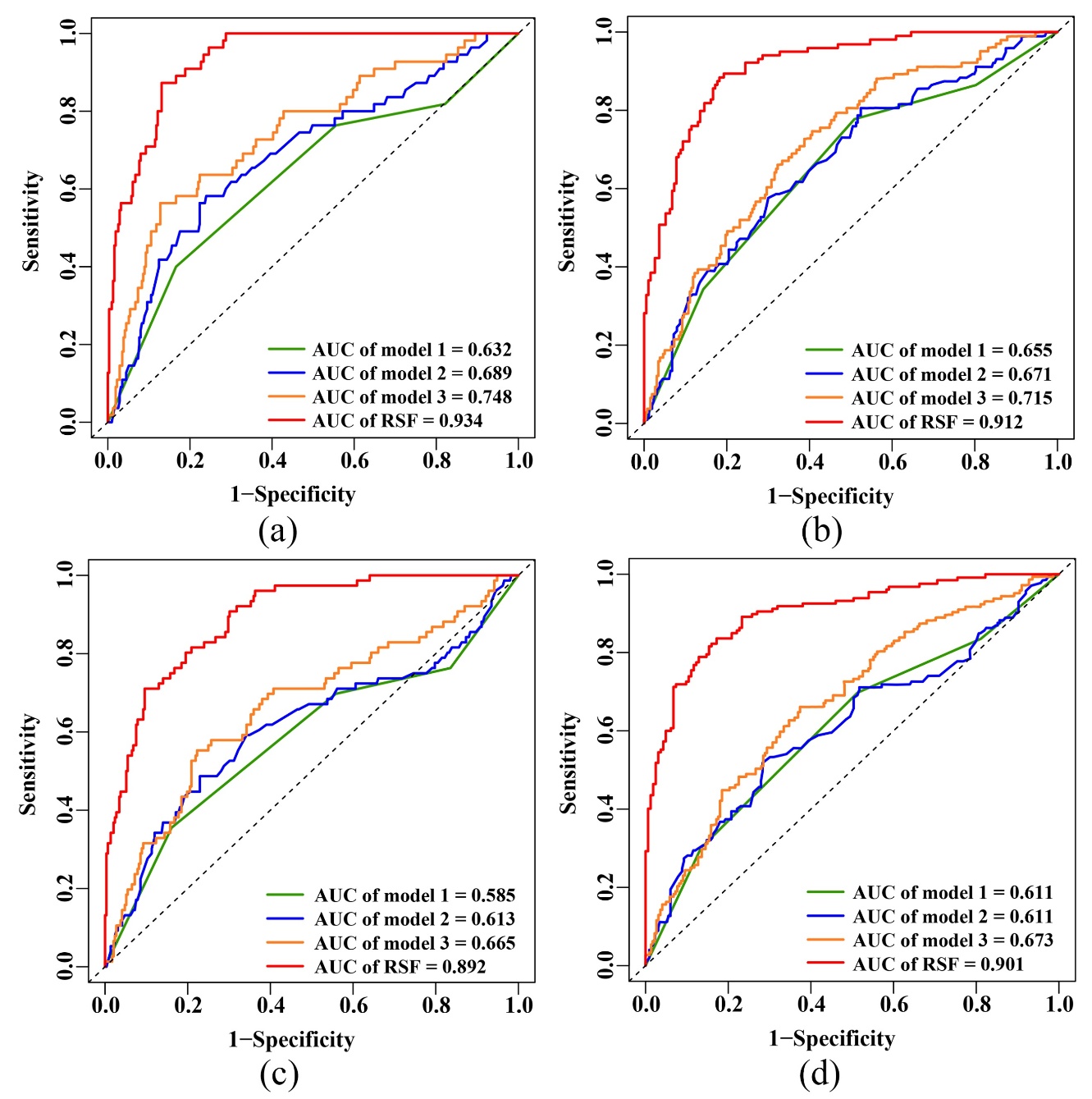


**Figure S3.** Calibration plots of RSF model in the training set. (a) 3-year primary endpoint. (b) 5-year primary endpoint. (c) 3-year secondary endpoint. (d) 5-year secondary endpoint. RSF, random survival forest.


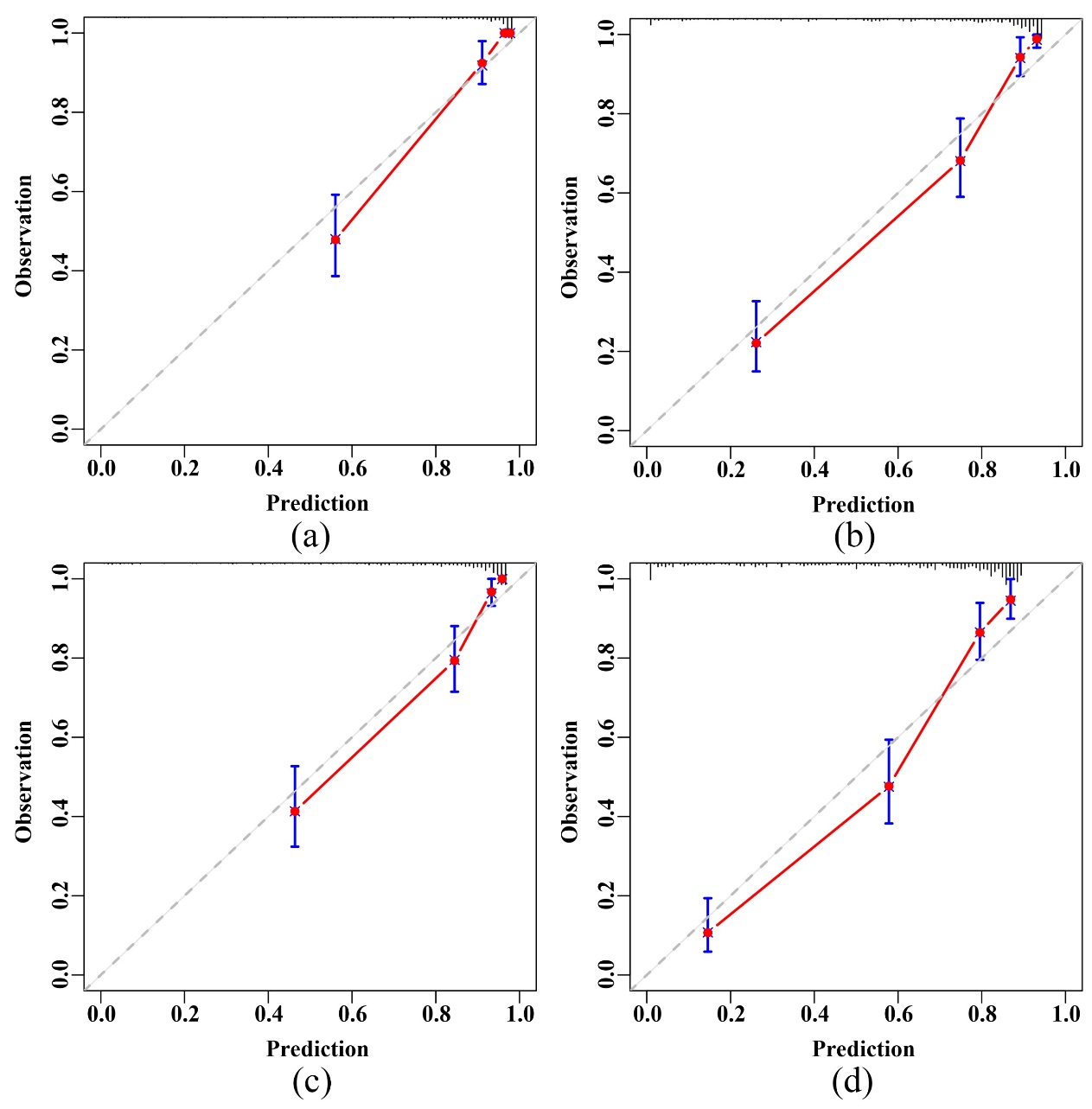


**Figure S4.** Kaplan–Meier curves of RSF model in the training set. (a) Primary endpoint. (b) Secondary endpoint. RSF, random survival forest.


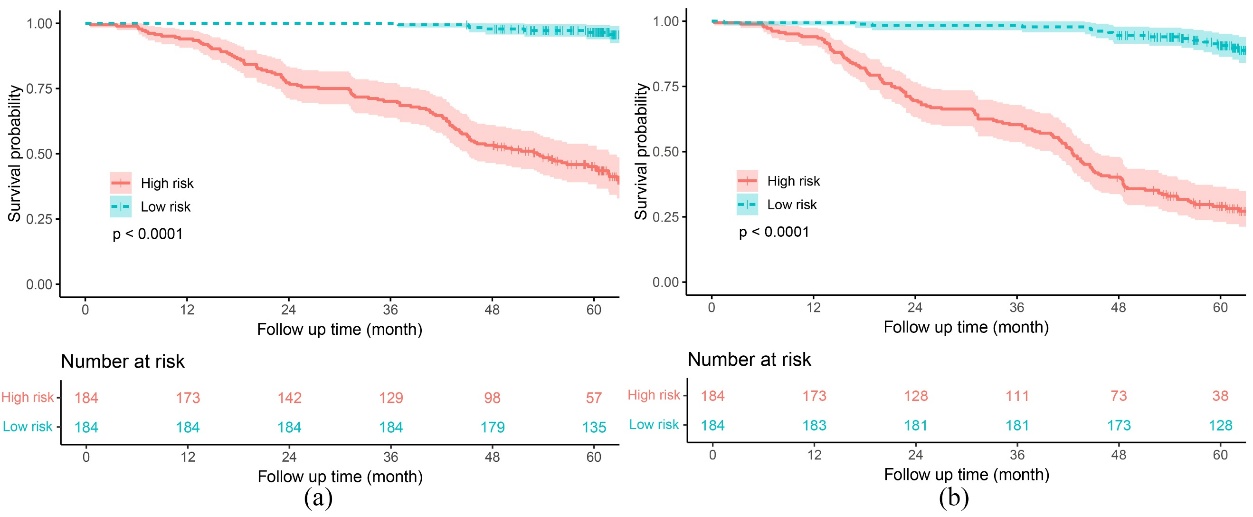

Supplement: Supplementary file 1 [file Datasheet1.docx]
